# Ovalbumin-Mediated Biogenic Synthesis of ZnO and MgO Nanostructures: A Path Toward Green Nanotechnology

**DOI:** 10.3390/molecules30051164

**Published:** 2025-03-05

**Authors:** Adriana-Gabriela Schiopu, Elena Andreea Vijan, Ecaterina Magdalena Modan, Sorin Georgian Moga, Denis Aurelian Negrea, Daniela Istrate, Georgiana Cîrstea, Mihai Oproescu, Şaban Hakan Atapek

**Affiliations:** 1 Faculty of Mechanics and Technology, Pitesti University Centre, National University of Science and Technology POLITEHNICA Bucharest, 110040 Pitesti, Romania; gabriela.schiopu@upb.ro; 2 Doctoral School Materials Science and Engineering, National University of Science and Technology POLITEHNICA Bucharest, Splaiul Independentei no. 313, Sector 6, 060042 Bucharest, Romania; elena_andreea.vijan@stud.sim.upb.ro (E.A.V.); calin_istrate_daniela@yahoo.com (D.I.); 3 Regional Center of Research & Development for Materials, Processes and Innovative Products Dedicated to the Automotive Industry (CRCD-AUTO), Pitesti University Centre, National University of Science and Technology POLITEHNICA Bucharest, 110040 Pitesti, Romania; sorin_georgian.moga@upb.ro (S.G.M.); aurelian.negrea@upb.ro (D.A.N.); georgiana.cirstea93@upb.ro (G.C.); 4 Faculty of Electronics, Communication and Computers, Pitesti University Centre, National University of Science and Technology POLITEHNICA Bucharest, 110040 Pitesti, Romania; mihai.oproescu@upb.ro; 5 Department of Metallurgical and Materials Engineering, Kocaeli University, 41001 Kocaeli, Türkiye; hatapek@kocaeli.edu.tr

**Keywords:** ovalbumin, eco-friendly, biogenic synthesis, nanostructures, ZnO, MgO

## Abstract

Sustainable and eco-friendly synthesis methods for nanoparticles are crucial for advancing green nanotechnology. This study presents the biogenic synthesis of zinc oxide (ZnO) and magnesium oxide (MgO) nanoparticles using ovalbumin, an abundant and non-toxic protein from egg white. The synthesis process was optimized by varying metal ion concentrations to control particle size and morphology. Characterization using ATR-FTIR, XRD, SEM, and UV-VIS confirmed the successful formation of uniform, well-crystallized nanoparticles with sizes ranging from 7.9 to 13.5 nm. ZnO nanoparticles exhibited superior antimicrobial efficacy against *Escherichia coli* and *Enterococcus faecalis*, while MgO nanoparticles showed enhanced potential environmental remediation. These findings highlight ovalbumin as a versatile agent for the green synthesis of ZnO and MgO nanomaterials, with promising applications in the medical, environmental, and optoelectronic fields. The results indicate that this biogenic method can serve as a sustainable proposal to produce nanostructured materials with diverse applications in the medical and environmental fields, such as eliminating pathogenic bacteria and purifying contaminated environments. Overall, this study significantly contributes to the development of sustainable nanomaterials and opens up new perspectives on the use of ovalbumin protein in the synthesis of multifunctional nanostructured materials.

## 1. Introduction

Nanostructures (NSs) have revolutionized scientific fields due to their unique properties, including small size, large specific surface area, high catalytic activity, and antibacterial and antimicrobial properties. The choice of elaboration method of NSs depends on various factors, including the desired properties of the nanoparticles, the scale of production, and the available resources. By carefully selecting the appropriate method, it is possible to synthesize metal oxide NSs with tailored properties for a wide range of applications. Among the variety of nanostructured oxides, ZnO and MgO are versatile and accessible materials, making them excellent examples of the potential of nanotechnology across various industries [1,2,3,4]. Zinc oxide (ZnO) and magnesium oxide (MgO) are versatile semiconductors with a wide band gap (3.37 eV-ZnO, 7.8eV-MgO) and excellent optical and electrical properties. Due to their nanoscale size, both ZnO and MgO nanoparticles (NPs) possess a high surface-to-volume ratio, which enhances their reactivity and catalytic activity in various applications and photocatalytic properties [5,6,7,8]. They can act as catalysts in various chemical reactions, including oxidation, reduction, and decomposition reactions. Thermal stability makes them suitable for high-temperature applications. Due to their shared properties, both ZnO and MgO nanoparticles find applications in various fields: catalysis, sensors, electronics, and biomedical applications. These oxides are also now used as an antibacterial agent against a wide range of bacteria species [9,10]. ZnO exhibits antibacterial properties both in the dark and under the action of light. Their mechanisms of action include the generation of reactive oxygen species (ROS), the release of ions, and the damage of the bacterial cell membrane. Both can be used in water or air purification, due to their ability to degrade toxic organic compounds. Also, both oxides can be used in combination to modify and improve the optoelectronic properties of devices.

To highlight the differences and similarities between zinc oxide (ZnO) and magnesium oxide (MgO), the comparison in Table 1 is organized based on their main properties and applications.

Top-down methods for metal oxide nanostructure synthesis involve breaking down bulk materials into smaller particles, often through physical or mechanical processes [11,12]. These methods are relatively simple and they can lead to variations in particle size and shape, as well as potential contamination from the processing equipment. Bottom-up methods involve building nanostructures from individual atoms or molecules. These methods offer greater control over particle size, shape, and crystallinity, making them highly desirable for many applications [5,13,14]. Previous studies have highlighted common synthesis methods for the two oxides, as well as the most used precursors, as shown in Table 2.

The conventional synthesis of metal nanoparticles often involves the use of toxic chemicals and entails high costs. Against the backdrop of global sustainability concerns, nanoparticle synthesis methods based on natural agents are being paid more attention. Biosynthesis, which involves the use of natural resources such as plants, microorganisms, or organic substances, has become a promising alternative to traditional chemical methods due to its low impact on the environment. Plant extracts are an affordable and rich source of phytochemical compounds such as phenols, flavonoids, and tannins, which facilitate the reduction in metal ions. For example, the use of *Portulaca oleracea* for the synthesis of ZnO nanoparticles has shown that the pH of the solution influences particle size and photocatalytic efficiency for the degradation of toxic dyes such as methyl orange [33].

Microorganisms, including fungi and bacteria, provide a robust platform for the synthesis of nanoparticles through intracellular or extracellular processes. Fungi such as *Fusarium oxysporum* produce nanoparticles by secreting enzymes that reduce metal ions and stabilize the resulting products [34]. This process is considered cost-effective and easy to scale [10]. The use of *Abelmoschus esculentus* and *Salvia officinalis* extracts for the synthesis of ZnO nanoparticles showed degradation rates of up to 86% for blue methylene in the first 90 min [30].

Distinguishing themselves from the use of plants, some studies focus on the use of other proteins in the biogenic synthesis of nanoparticles. For example, one review article discusses various methods for the synthesis and control of nanoparticle growth, highlighting the importance of proteins and other biomolecules in the formation and stabilization of nanoparticles [35].

Ovalbumin, a protein from egg white, is one choice, due to its availability and unique chemical properties, proving a promising agent for the controlled synthesis of nanoparticles. Egg white proteins have been used for the synthesis of silver (Ag) nanoparticles and metal oxides. Egg white acts as a reducing and stabilizing agent, forming uniform nanoparticles of about 20–25 nm, ideal for biomedical and energy applications [36,37].

Unlike plant extracts or microorganisms, which exhibit batch-to-batch variability due to genetic and environmental factors, ovalbumin is a single, well-characterized protein with a consistent structure. Ovalbumin has a well-defined molecular structure with functional groups (e.g., hydroxyl, carboxyl, and amine) that can effectively coordinate metal ions (Zn^2+^ and Mg^2+^), leading to the controlled nucleation and growth of ZnO and MgO nanostructures. This level of control is often challenging to achieve with plant extracts, which contain complex and variable compositions of biomolecules such as polyphenols, flavonoids, and alkaloids that can unpredictably influence particle morphology. Plant extracts contain a variety of organic compounds that may leave residual carbon and impurities after calcination, affecting the purity and functionality of the final nanomaterial. In contrast, ovalbumin undergoes a cleaner thermal degradation, leading to higher-purity ZnO and MgO nanoparticles with fewer organic contaminants. This allows for more reproducible synthesis conditions and uniform nanostructures, ensuring consistent size, shape, and characteristics, which are critical for applications in catalysis, sensing, and biomedicine. Egg white as source of ovalbumin is inexpensive, readily available, and easy to handle compared to plant extracts or microbial cultures, which require complex growth conditions, nutrient media, and precise environmental controls. This makes the ovalbumin-assisted synthesis method more scalable and economically viable for large-scale production.

Previous research focuses on ZnO nanoparticles’ egg white-assisted synthesis using zinc nitrate and eggs of different origin (chickens and ducks) [32]. Nitrate generates nitrogen oxide bonding during the synthesis process, requiring additional removal measures.

This research explores a biogenic approach that overcomes this limitation using zinc and magnesium acetate as sources of metals. Acetates are a less oxidizing salt compared to nitrates, which can favorably influence the synthesis reactions. Acetate, on the other hand, produces carbon dioxide and water upon thermal decomposition, which are more environmentally friendly and easier to manage. Also, zinc or magnesium acetate is more economically accessible and easier to store compared to nitrate. In the presence of egg white (which contains proteins), acetate can interact more gently, avoiding a sudden change in pH, which contributes to better control of the morphology of the nanoparticles.

## 2. Results and Discussion

### 2.1. Compositional Characterization

**Figure 1:**
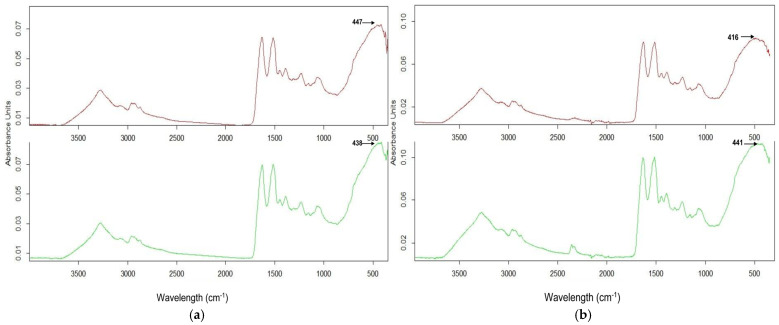
ATR-FTIR spectra of (**a**) ZnO NSs and (**b**) MgO NSs: 1M (magenta); 2M (green).

In the frame of current study, the ATR-FTIR spectra from Figure 1a show absorption bands at 438 cm^−1^ and at 447 cm^−1^, which had been attributed to ZnO the stretching frequency of ZnO bonds. The ATR-FTIR spectra from Figure 1b show absorption bands at 441 cm^−1^ and at 416 cm^−1^, which can be attributed to the stretching frequency of Mg–O bonds, as reported by as reported by Seghir et al. [29]. Compared to ZnO nanoparticles synthesized using Portulaca oleracea extract [34], where ZnO bonds were identified at 450 cm^−1^, the current study demonstrates slightly shifted peaks, which could be attributed to the ovalbumin-mediated synthesis process, leading to different bonding environments.

The band centered at 1630 cm^−1^ was ascribed to the presence of water molecules on the surface of oxides. Also, a peak at 1117 cm^−1^ was due to the re-absorption of water molecules from an ambient atmosphere. The presence of carboxyl chain residues in ovalbumin degraded during treatment at 120 °C is evidenced by the absorption bands at 1396 cm ^−1^, assigned to protein side-chain COO–. Carboxyl groups chelate metal ions and act as nucleation sites. Amino groups participate in metal ion binding and contribute to the reduction process. Thiol groups are strong reducing agents and can play a significant role in the reduction of metal ions. The amide I band from the C=O stretching vibrations of the peptide bond overlapped with the hydroxyl groups at 1630 cm^−1^. The band around 1550 cm^−1^ corresponds to N–H vibration and indicates the presence of the amide II band. The peak near 1070 cm^−1^ was attributed to C–O vibration. For all spectra, the absorption band at 3343 cm^−1^ was due to the stretching frequency of hydroxyl groups of absorbed water. The peaks around 2300–2400 cm^−1^ correspond to CO_2_ molecules from the air.

Table 3 exhibits the values of assignments of absorption bands of ZnO and MgO NSs synthesized with ovalbumin, starting from acetates after 4 h at 120 °C.

ATR-FTIR spectra of ZnO and MgO after 4 h reflect a predominance of inorganic components in the final composition.

**Figure 2:**
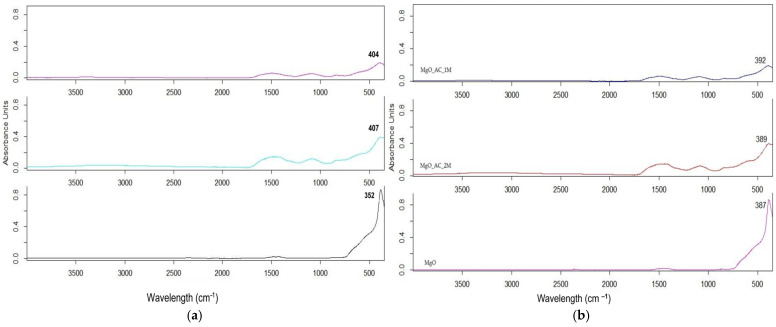
ATR-FTIR spectra of (**a**) ZnO NSs and (**b**) MgO NSs: 1M (magenta), 2M (green), and commercial (black) after calcination after 2 h at 550 °C.

For all samples calcinated at 550 °C for 2 h, the ATR-FTIR analysis consisted of a comparison with commercial ZnO and MgO powders of analytical purity, as shown in Figure 2. The pure ZnO presents an absorption band at 352 cm^−1^, while the pure MgO presents an absorption band at 387 cm^−1^ (black line in Figure 2).

Additionally, the disappearance of the broad O-H stretching band at 3343 cm^−1^ post-calcination at 550 °C indicates the successful removal of organic residues, which coincides with results obtained by Pachiyappan et al. [6], where the complete degradation of biogenic stabilizing agents was observed in ZnO and MgO hybrids. This confirms that the transformation of the hydroxide to the oxide phase occurred during the calcination process. The intensity of bands around 380–400 cm^−1^ suggests a well-crystallized ZnO NS and MgO NS, and the slight shift in the values in this region may be due to the interaction of ZnO with organic molecules (ovalbumin). Weak bands in the range of 1500–1000 cm^−1^ may be associated with functional groups of protein degradation, especially the C–O (carboxylic) and C–N (amide) stretching.

**Figure 3:**
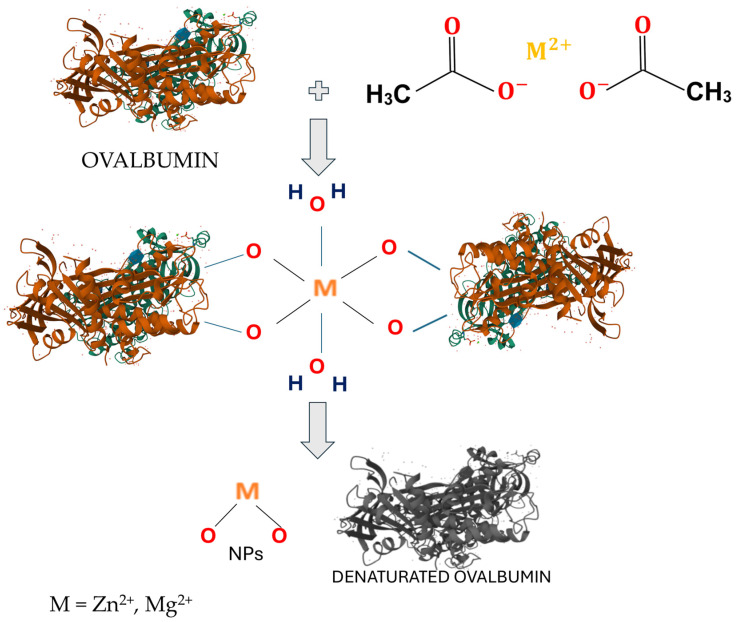
Mechanism of formation of metal oxides through biogenic synthesis using ovalbumin.

The mechanism of formation of metal oxides through biogenic synthesis is not fully known, even though there is previous research by Vijan et al. [33]. By analyzing the ATR-FTIR spectra after 4 h at 120 °C and after calcination at 550 °C for 2 h, we can propose the mechanism of ZnO and MgO formation via biogenic synthesis with ovalbumin. The mechanism of metal oxide synthesis with ovalbumin is presented in Figure 3. Ovalbumin, with its rich amino acid composition, can chelate metal (Zn and Mg) ions through various functional groups like carboxyl, amino, and thiol groups. This initial binding step is crucial for the subsequent reduction and nucleation processes.

The chelated metal ions on the ovalbumin surface can serve as nucleation sites for the formation of metal oxide clusters. The protein can stabilize the formed NS by capping its surface. This capping action prevents agglomeration and ensures the formation of discrete NSs.

A higher concentration of ions (2M) accelerates chelation, but may lead to faster saturation and competition for binding sites. Heating induces the denaturation of proteins, exposing additional sites for nucleation.

### 2.2. Crystalline Characterization

**Figure 4:**
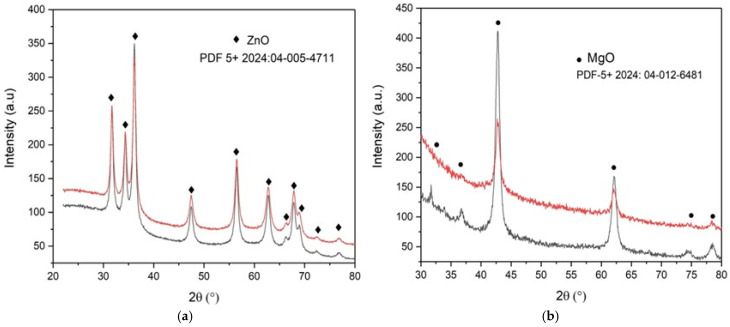
XRD patterns of (**a**) ZnO and (**b**) MgO NSs elaborated with ovalbumin, black 1M, and red 2M.

To assess the crystalline structure, XRD analysis was performed. All the diffraction peaks of the calcinated ZnO samples can be indexed to a hexagonal structure, which is very well matched with card number 04-005-4711, as presented in Figure 4a. Also, all the diffraction peaks of the calcinated MgO samples can be indexed to a cubic structure, which is very well matched with card number 04-012-6481, as presented in Figure 4b.

No characteristic peaks of any impurities are detected, which demonstrates that the product has high phase purity. Ovalbumin decomposes during calcination at 550 °C, leaving behind metal oxides. The decomposition rate impacts the transition from organic–metal complexes to pure crystalline metal oxides. A controlled heating rate (20 °C/min) ensures uniform conversion.

To understand the modification of the structural characteristics under biogenic synthesis using ovalbumin, the average crystallite size *D* (nm) of the nanoparticles was determined using the Williamson–Hall method:

(1) βcosθ=Kλ/D+4εsinθ

where *β* is the full-width-half-maximum (FWHM) of the prominent peaks in X-ray diffraction; *K* is the shape factor; *λ* is the X-ray wavelength (*λ* = 0.15418 nm); *D* is the particle size (D_W-H_); and *ε* is the lattice strain of ZnO and MgO NSs. The lattice parameters *a* and *c* (Å), cell volume V (Å^3^), and unit cell, volume u (Å) are also presented in Table 4. The values match well with the standard literature values [17,38,39].

The perfect wurtzite structure of ZnO has four-fold coordination with a hexagonal unit cell, having the fraction *c*/*a* = 8/3 = 1.633. The perfect halite structure of MgO is body-centered cubic geometry, with each magnesium ion (Mg^2+)^ being surrounded by six oxygen ions (O^2−^), and vice versa.

These values of a and c align well with the standard ZnO structure, confirming the expected hexagonal crystalline phase. The *c*/*a* ratio is approximately identical for both ZnO samples, which is very close to the ideal value for the wurtzite structure (1.633). This indicates well-ordered crystalline lattices with minimal distortion. The slight increase in crystallite size with increasing molarity suggests that ion concentration has an effect on nucleation kinetics, which is comparable to the conclusions of Hafef et al. [8], whereby ZnO-MgO composite structures showed increasing grain size with precursor concentration. The c/a ratio aligns with standard wurtzite ZnO values, supporting the minimal lattice distortion observed in the biogenic synthesis approaches of Stepan et al. [5].

The difference in crystallite size between MgO NSs arises from higher concentrations of the metal solution. The unit cell volumes are consistent between the two samples ZnO_1M and ZnO_2M NSs, suggesting that the change in molarity has minimal effects on the ZnO crystal structure. The slight increase in crystallite size and consistent lattice parameters indicate that the ZnO NSs are well crystallized, and that the higher molarity leads to slightly better crystallinity. The corresponding ZnO NS results can be compared with the results of Ahmed et al. [40]. Other researchers, such as Gherbi et al., obtained ZnO crystals two times bigger via Portulaca oleracea extract [33].

The MgO_1M NS exhibits a significantly larger crystallite size compared to the MgO_2M NS. This large reduction in crystallite size, with increased molarity, suggests that higher molarity significantly impacts nucleation and growth dynamics, possibly due to enhanced supersaturation during synthesis. A higher concentration of Mg (II) leads to faster nucleation and smaller crystal sizes. The values of the lattice parameters of MgO NSs match the standard MgO cubic structure. The slight increase in the unit cells of the MgO_2M NS may be attributed to lattice strain, or small variations in the synthesis process. The substantial decrease in crystallite size for MgO at higher molarity suggests that nucleation is favored by overgrowth at a 2M concentration. Both samples exhibit perfect cubic symmetry, and the structural integrity of MgO is maintained.

These results demonstrate the critical role of molarity in tailoring nanomaterial properties, such as crystallite size and structural parameters, which are crucial for their potential applications.

### 2.3. Morphological Characterization

**Figure 5:**
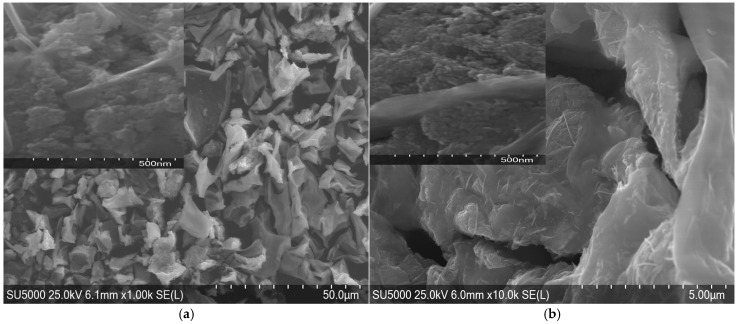
SEM morphologies of ZnO NSs elaborated with ovalbumin from Zn(II) (**a**) 1M and (**b**) 2M.

The nanoparticles of ZnO from Zn(II), 1M, exhibit a slightly elongated and rod-like shape, as observable in Figure 5a. There seems to be a relatively uniform size distribution with some agglomeration. The nanoparticles of ZnO from Zn(II), 2M, as shown in Figure 5b, display a more irregular and elongated morphology. There is a greater degree of agglomeration compared to Figure 5a. Compared to the research of Ahmed et al., the ZnO NSs obtained in this research are much more crowded, due to effect of a higher acetate concentration [40].

**Figure 6:**
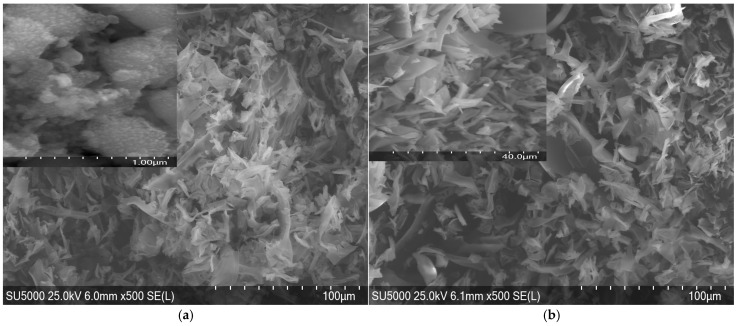
SEM morphologies of MgO NSs elaborated with ovalbumin from Mg (II) (**a**) 1M and (**b**) 2M.

MgO nanoparticles obtained with ovalbumin have a more regular shape, often semi-spheres, as shown in Figure 6. This may be due to a stronger interaction between magnesium ions and protein, which promotes a more isotropic growth of particles.

Similar morphologies were reported by Rode et al. [10], where biosynthesized ZnO nanoparticles showed anisotropic growth patterns due to variations in metal ion interaction with stabilizing biomolecules. The aggregation observed at higher ion concentrations aligns with previous reports from Selvi et al. [16], suggesting that increased nucleation sites in the presence of a higher precursor concentration led to densely packed nanostructures.

The higher concentration of Zn(II) and Mg(II) ions might have led to faster nucleation and growth, resulting in the observed changes in morphology and agglomeration. Faster nucleation and growth were due to the higher reactivity of Zn^2+^ ions, leading to elongated structures. Slow nucleation with isotropic growth causes semi-spherical particles of MgO.

For both ZnO and MgO, increasing the concentration of metal ions (from 1M to 2M) leads to a more irregular morphology and an increase in the degree of agglomeration.

The presence of ovalbumin in the synthesis process significantly influences the morphology of the particles, acting as a trapping agent and controlling crystal growth. In both cases, we observe a tendency towards the formation of agglomerates, especially at higher concentrations of metal ions. Slower nucleation with isotropic growth results in semi-spherical particles.

The role of ovalbumin as a morphological directing agent can be assumed from the well-defined shapes observed in the SEM images. This behavior aligns with the results of Lu et al. [36], who demonstrated that protein-mediated synthesis promotes isotropic growth, leading to defined structural features in metal oxide nanoparticles.

### 2.4. Optical Characterization

**Figure 7:**
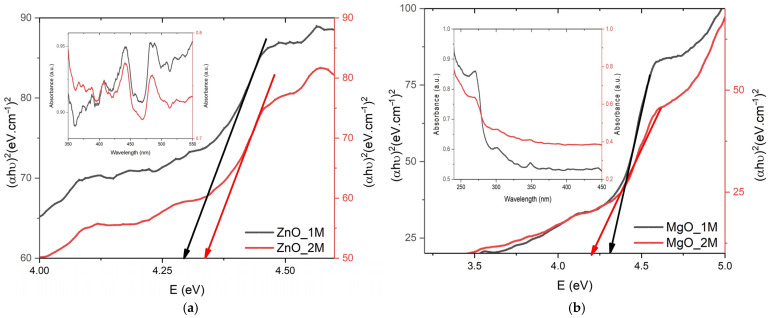
UV-VIS spectra and Eg plot of ZnO and MgO NSs elaborated with ovalbumin at (**a**) 1M and (**b**) 2M.

The UV-VIS spectra were measured in the range of 200–1000 nm. In Figure 7a show a clear increase in the Eg value for the ZnO_2M NS, consistent with a better-ordered crystal structure and a reduction in defects. The increase in Eg is attributed to an improvement in NS quality rather than quantum size effects, given the similar sizes of the crystallites.

The UV-VIS spectra in Figure 7b and Eg values show that the MgO_1M NS exhibits optical properties closer to those of the bulk material, with better crystalline quality and fewer defects. MgO_2M, on the other hand, exhibits a reduced Eg and an absorption shifted towards longer wavelengths, due to the decrease in crystallite size and the increase in structural defects.

The calculated Eg value (4.29 eV) of the ZnO_1M NS is higher than the typical theoretical value for ZnO (~3.37 eV in bulk). This increase indicates quantum effects, which are consistent with the small crystallite size (~12 nm). Quantum size effects appear for particles below 20 nm, and shift the absorption edge to shorter wavelengths, a process called blue shift. A slight increase in the crystallite size of the ZnO_2M NS (12.5 nm compared to 12.02 nm) is observed, but the difference is small, as shown in Table 5. The increased Eg value to 4.34 eV suggests a slightly higher electronic separation, possibly due to increased purity. This shift corresponds to the quantum confinement effects reported by Haque et al. [39], where smaller ZnO nanoparticles exhibited an increased bandgap energy due to reduced electron–hole recombination.

MgO typically exhibits absorption in the UV region due to its broad band energy (~7.8 eV in bulk). The measured Eg value (4.31 eV) of the MgO_1M NS is lower than the theoretical one, suggesting structural changes due to the influence of ovalbumin. The reduced Eg value (4.20 eV compared to 4.31 eV) of the MgO_2M NS can be attributed to the additional effects induced by the particle sizes. The same effect was observed in studies by Eissa et al. [28]. The discrepancy between theoretical and measured Eg values suggests that the interaction of ovalbumin with MgO during synthesis alters electronic transitions, as was also reported by Maruthai et al. [15]. The absorption is shifted to lower energies (red shift).

The higher Eg values for ZnO (compared to the theoretical ~3.37 eV) indicate good crystalline quality and quantum effects. The Eg value for the MgO NS is lower than the theoretical one (~7.8 eV), indicating the possible effects of functional groups of amino acid chains on the NS surface. 

The UV-VIS spectra confirm the characteristic absorption of ZnO and MgO nanoparticles, influenced by concentration and size.

### 2.5. Characterization of Antibacterial Properties

ZnO_1M inhibited *E. coli* growth completely after 5 h of contact, as shown in Table 6. However, it had a limited effect on *E. faecalis*, with only a slight reduction in the colony-forming units (CFUs). ZnO_2M exhibited similar results to 1M ZnO, completely inhibiting *E. coli* growth after 5 h, but showing a less pronounced inhibition of *E. faecalis*. This performance aligns with studies by Ogunyemi et al. [27], whereby ZnO nanoparticles synthesized using green methods exhibited significant bacterial suppression through reactive oxygen species (ROS) generation. The mechanism involves the disruption of bacterial membranes via ROS, ion release, and electrostatic interactions, as reported by Seghir et al. [29].

MgO_1M demonstrated a moderate inhibitory effect on both bacteria, with a significant reduction in the CFUs after 5 h of contact. MgO_2M showed a slightly stronger inhibitory effect than 1M MgO, with a greater reduction in the CFUs for both bacteria after 5 h. The results that are in agreement with Bachhav et al. [19], whereby MgO showed selective inhibition against Gram-positive bacteria. The variation in efficacy between ZnO and MgO can be attributed to differences in ROS production and surface charge density, as discussed by Hafef et al. [8].

Following the contact of ZnO and MgO NSs with the bacterial suspension containing *Escherichia coli* and *Enterococcus faecalis* for ~5 h at 25 °C, good results on the inhibitory action of these NS oxides on bacterial growth were obtained. The longer the contact time between the oxides and the bacteria, the greater the inhibitory action on the bacterial growth was.

The antibacterial properties are in direct connection with the small band gaps of NS oxides, because the band gap of a semiconductor influences the photon energy required to excite electrons and generate ROS. Nanoparticles with a smaller band gap can be excited by a wider range of wavelengths of light, which gives them greater potential to generate ROS and have greater antibacterial activity.

## 3. Materials and Methods

### 3.1. Materials and Reagent for Synthesis

Free-range chicken egg whites were collected. These served as sources of ovalbumin for ZnO and MgO NP synthesis. The synthesis of ZnO and MgO nanostructures consisted of the dropwise addition of 50 mL of Zn(II) and Mg(II) solution into 50 mL of ovalbumin foam from free-range chicken eggs.

Zinc acetate dehydrate a.p., CAS-5970-45-6, from Sigma Aldrich and magnesium acetate tetrahydrate a.p., CAS-16674-78-5, from Roth were used as sources of metallic ions. The Chromocult Coliform agar, Slanetz and Bartley agar, media for microbiology, and sterile distilled water were used for bacterial cultures during antibacterial testing. *Escherichia coli* ATCC25922 and *Enterococcus faecalis* ATCC19433 were used to evaluate the antibacterial efficacy of the biogenically synthesized NSs.

### 3.2. Synthesis of ZnO and MgO Nanoparticles with Ovalbumin

The ability of egg white proteins to form a gel upon heating and their water-binding and emulsifying capacity are important functional properties. Ovalbumin is the essential protein of egg white. It has a unique sequence located in the middle of the protein chain, rather than at the N-terminus, as in most proteins. In the literature, different terminologies are used for ovalbumin: native (N-), stable (S-), and intermediate (I-) ovalbumin. N-ovalbumin can be converted into S-ovalbumin through the formation of an intermediate (I-) [41]. All these species can aggregate. The ovalbumin can self-assemble into various nanostructures, such as fibers, spheres, and capsules. The specific arrangement of amino acids in the polypeptide chain determines the unique three-dimensional structure of ovalbumin and its functional properties. Ovalbumin aggregation is generally triggered by a heat-induced conformational change in the protein, or by the binding of metal ions that affect the folded structure [37,38,41]. Metal ions can be encapsulated within ovalbumin using various techniques, such as ionic cross-linking or electrostatic interactions.

Thus, ovalbumin can serve as a template for ZnO and MgO NS synthesis through various mechanisms:

1. Zn and Mg ions can coordinate with the functional groups present in ovalbumin, such as amino and carboxyl groups. Zn and Mg ions can be incorporated into these self-assembled structures, followed by calcination to remove the organic template and obtain metal oxide nanostructures with controlled morphology.
2. The protein can act as a reducing agent, converting metal ions into metal nanoparticles. The resulting metal nanoparticles can be further oxidized to form metal oxide nanoparticles.

**Figure 8:**
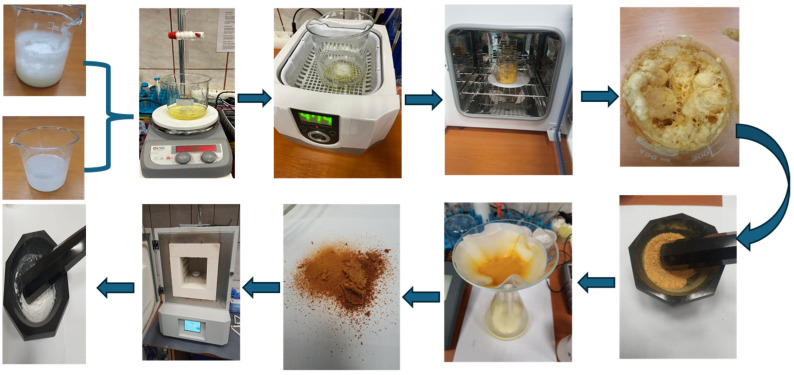
Elaboration process flux.

The elaboration process flux presented in Figure 8 outlines each stage, from the initial solution preparation to the final powdered nanostructure product.

In total, 50 mL of egg white is separated from the yolk and collected in a beaker. The egg white serves as a natural source of ovalbumin, which acts as a stabilizing and templating agent. The 50 mL acetate solution of 1M and 2M concentration is added dropwise to the egg white foam. For each mL of acetate added, the pH is measured to identify the turning point corresponding to the formation of the ovalbumin–metal complex. This mixture is stirred without heating to ensure thorough mixing, forming a homogeneous solution. The mixture is subjected to sonication using an ultrasonic bath. This step helps to uniformly disperse the precursor within the egg white matrix and facilitates interaction between the ovalbumin and metal ions. After all the acetate solution has been added, the metal–foams are formed. The drying of foam was performed in a Biobase oven at 120 °C for 4 h. This removes the water content and converts the mixture into a solid foam structure. Yellow initial powders were obtained after grinding the dyed foam. The amount of obtained dried powders is presented in Table 7, in association with the amount of metallic acetate. These powders were again washed with deionized water and dried for 2 h at 120 °C.

After dying, a Mikrotest MKF-05, Ankara, Türkiye, muffle oven was used for the calcination of powders at 550 °C, at a controlled rate of 20 °C/min, for 2 h to ensure the complete decomposition of the metal–ovalbumin complex. Cooling was performed at 20 °C/min to stabilize the properties. After calcination, a fine white powder was obtained.

The research was conducted at different concentrations of Zn(II) and Mg(II) to study whether a higher concentration led to a higher nucleation rate in ovalbumin foam, resulting in a larger number of smaller crystallites.

**Figure 9:**
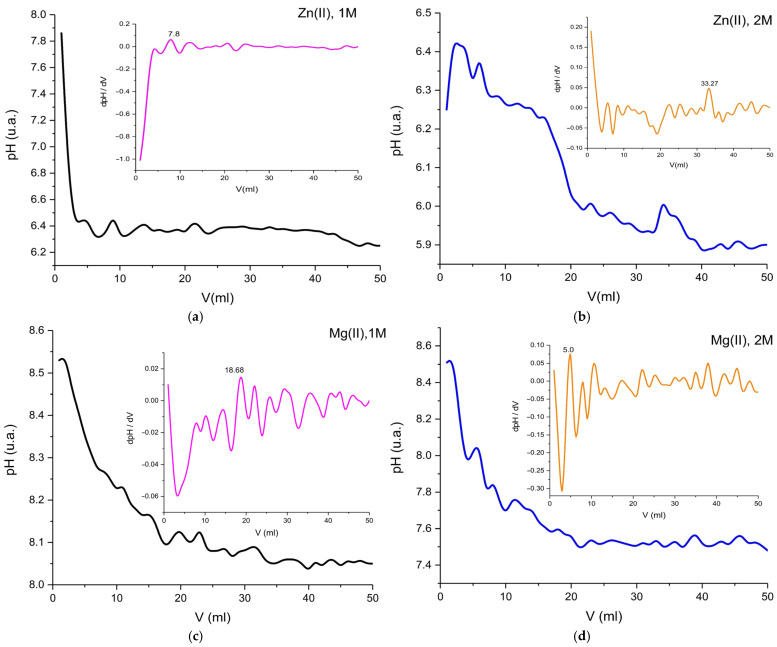
Graphical representation of the pH = f(V) variation for Zn(II) solutions ((**a**) 1M and (**b**) 2M) and Mg(II) ((**c**) 1M and (**d**) 2M).

The addition of Zn(II) or Mg(II) might initially cause a change in pH, due to the interaction of the metal ions with the functional groups on the ovalbumin. The experiments were conducted at room temperature. The initial decrease in pH suggests that Zn(II) and Mg(II) displace protons from the protein. As more metal ions were added, the pH stabilized. This indicates a buffering region, as shown in Figure 9.

Amino acid residues from ovalbumin with ionizable groups (like carboxylic acids and amines) can act as buffers, resisting changes in the pH. This could indicate that the metal ions bind to specific sites on the ovalbumin, forming stable complexes, where the pH reaches a point at which the complex becomes insoluble.

The heat-induced denaturation of foams was studied using ATR-FTIR. Calcination is the critical step where the organic template decomposes, leaving behind metal oxide nanostructures.

### 3.3. Methods of Characterization of ZnO and MgO Nanostructures

ZnO and MgO’s crystalline structures were assessed via XRD using the Rigaku Ultima IV diffractometer, Japan. The scan speed was set at 2°/min in Bragg–Brentano geometry using CuKα radiation (λ = 0.154 nm) at 45 kV and 40 mA, along with a one-dimensional D/teX Ultra detector equipped with a graphite monochromator. The X-ray diffractograms were recorded over a measuring range of 2θ: (28–105°), with a step size of 0.05°. The XRD spectra were analyzed using the PDXL 2 program, and crystalline compounds were identified using the PDF 5+ database.

To assess the role of ovalbumin, during the drying process, the samples were subjected to Attenuated Total Reflectance–Fourier Transform Infrared Spectroscopy analysis using a Tensor 27 spectrometer, Bruker, Germany, in the range of 350–4000 cm^−1^, with a resolution of 4 cm^−1^.

An Ocean Optics HR2000+ spectrometer was used to measure and record the UV–Vis absorption spectra of nanoparticle solutions. The optical energy band of a semiconductor was obtained using the following equation:

(2) $$\alpha =\frac{k{\left(h\vartheta -Eg\right)}^{n/2}}{h\vartheta }$$

where *k* is a constant, α is the absorption coefficient, *Eg* is the energy band gap, and *n* is 1326 for a direct energy band gap. The Eg can be estimated from the Tauc plot of (αhυ)^2^ versus the energy of a photon (hυ) [30]. The intersection of the tangent on the Tauc plot gives a direct band gap for n = 1.

The shape and size of ZnO and MgO NS were analyzed using Scanning Electron Microscopy (SEM) (SU 5000 microscope, Hitachi, Tokyo, Japan). The samples were fixed on carbon tape. The morphology was analyzed at different magnifications at 25 kV acceleration voltage.

Nanoparticles can interact directly with the bacterial cell membrane, disrupting its integrity and leading to the loss of the cellular contents. Nanoparticles with a sharp or irregular shape can more easily penetrate the bacterial cell membrane. Also, the absorption of light by nanoparticles can lead to a local increase in temperature, which can denature bacterial proteins and damage the cell membrane.

The loops with bacterial load were hydrated, each in 1 mL of sterile distilled water, for 30 min at 37 °C, while sterile loop passages were made on the non-selective medium, Tryptic Soy agar, and incubated at 37 °C for 24 h. From these subcultures, work crops were carried out in the same way. From the working culture, a colony was unloaded into 10 mL of sterile distilled water, to determine the approximate number of bacteria. A McFarland densitometer was used. Knowing that a colony had a bacterial load of approximately 1.5 × 10^8^ CFU/mL, decimal dilutions up to 10^−6^ were performed for *Escherichia coli* and 10^−6^ for Enterococus faecalis, followed by testing each dilution to establish the number of bacteria of interest and the number of developed colonies that could be read on the culture medium. The culture media used were the Chromocult Coliform agar and Merck’s Slanetz and Bartley agar. They were prepared according to the manufacturer’s instructions, with a concentration of 41.5g/L for the Slanetz–Bartley agar and 26.5 g/L for the Chromocult Coliform agar medium, sterilized in a water bath, allowed to reach a temperature of about 47–50 °C, and then poured into Petri dishes. A quantity of 2 mg of each NS oxide was weighed and placed in a test tube containing 9 mL of sterile distilled water and 1 mL of bacterial suspension.

The positive control is represented by the sample with 9 mL of sterile distilled water and 1 mL of bacterial suspension. The number of microorganisms estimated to grow on the positive control medium was approximately 300 *Escherichia coli* colonies/1mL sample and 50 enterococcal colonies/1 mL filtered sample.

Before being immersed in the test tubes with 10 mL of artificial sample, the NS oxides were sterilized under the UV lamp in the microbiological niche for 15 min.

The test tubes in which the NS oxides were introduced were incubated in the thermostat at a temperature of 25 °C, for about 30 min, to observe their bactericidal and bacteriostatic action on the microorganisms. After this time, from each test tube, 1mL of sample was filtered through the filter membranes (with a pore size of 0.45 μm) for the determination of *E. coli* and *E. faecalis*, and the rest of the samples with microorganisms and oxides of Zn and Mg were maintained for up to 5 h under the same environmental conditions (25 °C, in the dark). The membranes were placed in Petri dishes on specific culture media for the development of typical colonies of *Escherichia coli* and *Enterococcus faecalis*. Petri dishes were incubated in thermostats at 37 °C for 24 h for Coliform bacteria (*E. coli*) and 48 h for *Enterococcus faecalis*.

## 4. Conclusions

The biogenic synthesis approach offers several advantages over traditional chemical methods, including eco-friendliness, biocompatibility, and control over NS size and shape.

The synthesis of ZnO and MgO NSs using ovalbumin allows to produce materials with varied morphologies, which can be controlled by modifying the concentration of metal ions. The specific mechanism can be influenced by factors such as metal ion concentration and pH.

Characterization techniques like ATR-FTIR, XRD, SEM, and UV-VIS are used to analyze the surface chemistry, size, crystallinity, and shape of the synthesized ZnO and MgO NSs. The study demonstrates the ability to control particle size and morphology through variations in metal ion concentration, enabling tailored properties for specific applications. The SEM analysis revealed the tendency of ZnO to form elongated structures and of MgO to form semi-spherical shapes, influenced by ovalbumin.

The study highlights the versatility of ovalbumin as a biogenic agent, leveraging its functional groups for efficient metal ion chelation and stabilization during nanoparticle synthesis. By understanding and optimizing the synthesis process, researchers can tailor nanomaterial properties for diverse applications, including antibacterial treatments, photocatalysis, and environmental purification. Furthermore, the use of acetate precursors instead of traditional nitrates offers an eco-friendlier alternative, reducing harmful byproducts and improving overall sustainability.

ZnO_1M NSs biogenically synthesized with ovalbumin are more suitable for applications requiring higher photocatalytic activity in the UV range, or where surface sensitivity is crucial (sensors or antibacterial applications), while the ZnO_2M NSs are more suitable for advanced optoelectronic applications, durable devices, and UV protection, due to their higher Eg values and better crystalline quality.

MgO_1M NSs biogenically synthesized with ovalbumin are suitable for industrial applications where stability is a priority, such as refractory materials, gas adsorption, and optical layers. MgO_2M NSs are ideal for applications requiring intense surface interactions, such as catalysis, water treatment, and antibacterial applications.

In conclusion, the findings of this study pave the way for the broader adoption of biogenic methods in nanotechnology. Ovalbumin-mediated synthesis presents a scalable and sustainable solution for producing high-quality ZnO and MgO nanoparticles and addressing global challenges in the environmental and biomedical fields. This work contributes to the development of greener synthesis pathways, promoting innovation in the creation of nanostructured materials for a sustainable future.

## Figures and Tables

**Table 1:** Main properties and applications of ZnO and MgO nanoparticles.

Characteristic	ZnO	MgO	Strengths	Reference
Structure and physical properties				
Crystal Structure	Hexagonal (wurtzite, zincite)	Cubic (halite)	ZnO: More suitable for optical devices due to the wurtzite structure. MgO: Excellent thermal stability due to its cubic structure.	[6,7,8]
Density	~5.61 g/cm^3^	~3.58 g/cm^3^	MgO: Lighter material, useful in porous material application.	
Resistance to High Temperatures	Moderate (~1975 °C melting point)	Excellent (~2852 °C melting point)	MgO: Ideal for refractory and thermal insulator applications.	
Optical and Electronic Properties				
Bandgap	3.37 eV	7.8 eV	ZnO: Ideal for photocatalysis and UV sensors. MgO: Excellent performance as an electrical insulator.	[9,11,12,13,14]
Conductivity	Semiconductor	Insulator	ZnO: Enables use in electronic and optoelectronic devices.	
UV Light Absorption	Excellent	Limited	ZnO: Superior performance in UV applications (e.g., sunscreens, photocatalysis).	
Catalytic properties				
Photocatalysis	Excellent due to UV absorption	Limited	ZnO: Used for degrading organic compounds and environmental purification.	[15,16,17]
Adsorption	Moderate	Excellent for gasses	MgO: Superior performance in CO_2_ adsorption and removal of gaseous pollutants.	
Antimicrobial Activity				
Antibacterial	Active in a broad spectrum	Active in a broad spectrum	Both: Offer antimicrobial activity, but ZnO may be more effective against Gram-negative bacteria.	[9,10,13]
Toxicity	Non-toxic at low concentrations	Very safe for skin	MgO: Preferred in applications where skin safety is a priority.	

**Table 2:** Common synthesis methods for ZnO and MgO nanoparticles.

Method	Description	Precursors for ZnO	Precursors for MgO	Reference
Ablation	Solar concentrated energy on metal-oxide precursors.	Zinc oxide	Magnesium oxide	[15,16,17]
Chemical Vapor Deposition (CVD)	Chemical reaction between gaseous precursors, depositing solid material on a substrate.	Zinc acetate, diethylzinc, dimethylzinc	Magnesium chloride, magnesium nitrate	[4,18]
Sol–Gel Method	Formation of a sol, gelation, and heat treatment.	Zinc acetate, zinc nitrate	Magnesium nitrate, magnesium chloride	[19,20,21]
Hydrothermal	Reactions in a sealed autoclave under high temperature and pressure.	Zinc acetate, zinc nitrate	Magnesium nitrate, magnesium chloride	
Microwave-assisted synthesis	Microwave irradiation accelerates chemical reactions.	Zinc acetate, zinc nitrate	Magnesium nitrate, magnesium chloride	[22,23]
Electrospinning	Electrospinning polymer solution into fibers, followed by calcination.	Zinc acetate, zinc nitrate	Magnesium nitrate, magnesium chloride	[13,24,25,26]
Biosynthesis	Utilizes biological agents for eco-friendly synthesis.	Zinc nitrate, zinc acetate	Magnesium nitrate, magnesium chloride	[6,27,28,29,30,31,32]

**Table 3:** ATR-FTIR peak assignments of ZnO and MgO NSs after 4 h at 120 °C.

Spectral Range (cm^−1^)	Vibrations for ZnO NSs	Vibrations for MgO NSs	Interpretation
350–500 cm^−1^	Zn–O stretching and bending (438, 447 cm^−1^)	Mg–O stretching and bending (416, 441 cm^−1^)	Characteristic vibrations of the metal–oxide bond.
1000–1500 cm^−1^	Organic contributions: C–O and C–N stretching (weak/moderate)		Contribution from carboxyl (COO–) or amide groups in proteins (ovalbumin).
1500–1700 cm^−1^	Amide I (C=O, 1650 cm^−1^) and Amide II (N–H, 1550 cm^−1^)		Amide bonds in denatured proteins (ovalbumin).
2500–3500 cm^−1^	Broad O–H and N–H stretching (hydrogen bonds, hydration)		Hydroxyls on the surface of the materials or residual adsorbed water.
3000–3500 cm^−1^	O–H stretching (wide)		Hydroxyl groups from water or proteins (hydrogen bonded to molecules adsorbed on ZnO and MgO NSs).

**Table 4:** XRD data of ZnO and MgO NSs.

Sample	*D* (nm)	*a* (Å)	*c* (Å)	*c*/*a*	V (Å^3^)	u (Å)
ZnO_1M	12.0 ± 1.7	3.2536	5.2126	1.6021	47.79 ± 0.2	3.6289
ZnO_2M	12.5 ± 0.4	3.2558	5.2167	1.6022	47.88 ± 1.8	3.6312
MgO_1M	7.9 ± 0.3	4.2190	4.2190	1	75.10 ± 0.4	4.2190
MgO_2M	13.5 ± 0.3	4.2230	4.2230	1	75.30 ± 0.7	4.2227

**Table 5:** Average crystallite size correspondence with Eg of NS oxides.

Sample	Average Crystallite Size (nm)	Eg (eV)
ZnO_1M	12.02 ± 1.7	4.29
ZnO_2M	12.5 ± 0.4	4.34
MgO_1M	7.88 ± 0.3	4.31
MgO_2M	13.5 ± 0.3	4.20

**Table 6:** The antibacterial properties of the NS oxides.

Sample	Contact Time < 1 h	Contact Time 5 h	Contact Time < 1 h	Contact Time 5 h
	*Escherichia coli* CFU/1 mL	*Escherichia coli* CFU/1 mL	*Enterococcus faecalis* CFU/1 mL	*Enterococcus faecalis* CFU/1 mL
Negative control sample	0	0	0	0
Positive control	~300	~300	44	29
ZnO_1M	~280	0	37	14
ZnO_2M	~270	0	36	8
MgO_1M	~300	89	43	7
MgO_2M	~300	87	34	20

**Table 7:** The amount of metallic acetate and of elaborated initial dried powders.

Sample	Metallic Acetate (g)	Dried Powders (g)
ZnO_1M	9.17	3.36
ZnO_2M	18.35	3.53
MgO_1M	10.72	3.42
MgO_2M	21.44	3.76

## Data Availability

Data are contained within the article.
